# Development in the comprehension of phonetically reduced spoken words

**Published:** 2025

**Authors:** Caroline Beech, Megan Shelton, Daniel Swingley

**Affiliations:** Department of Psychology, 425 S. University Ave., Philadelphia, PA 19104 USA; Department of Psychology, 425 S. University Ave., Philadelphia, PA 19104 USA; Department of Psychology, 425 S. University Ave., Philadelphia, PA 19104 USA

**Keywords:** language acquisition, pronunciation variability, word recognition, reduction

## Abstract

The speech young children hear is highly variable. For example, reduced pronunciations, where some sounds in the canonical pronunciation are naturally dropped or altered, are common even in speech to children. The present study employed a new story-guided looking method (a variation on language-guided looking) to create felicitous conditions for testing young children’s recognition of reduced pronunciations of familiar words. Experiment 1 (18–24 months, n=32) found that toddlers succeeded at recognizing clear pronunciations, but failed to recognize reduced pronunciations, even in repetition trials when target words were preceded by a clear mention of the same word in the previous sentence. In Experiment 2, 3-year-olds (35–39 months, n=17 out of 44 pre-registered, ongoing) succeeded at recognizing reduced pronunciations, and benefited from preceding repetition. Overall, these results demonstrate a powerful new method for studying children’s language comprehension under more naturalistic conditions, and highlight an important psycholinguistic development over the 2–3 year span.

## Introduction

Quantitative models of language acquisition often implicitly assume that all speech is equally interpretable to children as input for learning. Each time a parent says a word, it is modeled as an opportunity to update phonotactic probabilities, hypothesize an intended referent, or notice a morphological marker, and it is often assumed that different instances of the same word can be recognized as such and grouped together. However, normal speech is highly variable. Speakers’ productions are not always clear, and the pronunciation of a given word varies even within the speech of one speaker, whether in adult conversation (e.g., Greenberg, 1999; Johnson, 2004; Warner, 2019), or child-directed speech (e.g., Bernstein Ratner, 1984; Buckler et al., 2018; Khlystova et al., 2023; Lahey & Ernestus, 2014). Indeed, many instances of words in spontaneous speech are so *reduced*, or weakened acoustically (shorter duration, lower intensity, no prominent pitch movement) and phonetically (e.g., non-schwa vowels pronounced more like a schwa, voiceless consonants pronounced more like voiced ones, omission of certain consonants or unstressed vowels, mushy realization of adjacent sounds), that they are unrecognizable when presented in isolation (e.g., Bard & Anderson, 1983; Pollack & Pickett, 1963). This raises important questions about how well young children, who are still learning the language, can interpret the reduced pronunciations characteristic of normal spontaneous speech.

For adults, making sense of reduced pronunciations poses little noticeable difficulty. Most people are not even aware of just how much reduction there is (Warner, 2019), even though reduced forms sometimes elicit a small processing cost in single-word recognition tasks in the lab (e.g., Tucker, 2011). Theories of (adult) speech perception and spoken word recognition offer a range of proposals to explain how listeners recognize reduced pronunciations: by matching phonetically close variants to known lexical items (Magnuson & Crinnion, 2022) (since these have already been learned), by storing multiple pronunciations for words with high-frequency pronunciation variants (Ranbom & Connine, 2007), and by making use of the surrounding phonological and lexical context, as well as the discourse context more broadly (Brouwer et al., 2013; Ernestus et al., 2002). These explanations seem plausible for adults, who have already learned the language they are trying to comprehend, but what about young children, who are still learning the words of the language, and discovering how to properly attribute aspects of the acoustic signal to different sources? While some research has investigated children’s ability to recognize words when produced by different talkers (e.g., Bulgarelli & Bergelson, 2022), in different accents (e.g., Best et al., 2009; Schmale et al., 2010), or with different tones of voice (e.g., Singh et al., 2004), little previous research has explored children’s ability to handle the pronunciation variability introduced by reduction.

Traditional methods for assessing young children’s word recognition focus on what children can do under ideal conditions, removing many of the complexities of children’s natural language environments. In typical “looking while listening” or “language-guided looking” experiments (Fernald et al., 1998; Swingley, 2009), children see a sequence of independent trials featuring simple isolated images that are explicitly labeled using hyper-clear ‘experimenter speech’. For instance, children might see an isolated image of a dog on one side of a computer screen and an isolated image of a baby on the other side, while listening to the phrase “Look at the dog!”. Looking time to the target image (the dog) rather than the distractor image (the baby) is taken to index children’s recognition of the word. This basic procedure, sometimes with manipulations to the speech or images to test if infants still recognize the correspondence, has been widely used for assessing children’s underlying *knowledge* about what words mean and how they sound (e.g., Beech & Swingley, 2023; Bergelson & Aslin, 2017). However, it is less well suited for answering questions about children’s *performance* under more naturalistic conditions.

In the present study, we developed a new “story-guided looking” method, a variation on the language-guided looking method that embeds the same fixation procedure (measuring children’s looking to one of two pictures given a sentence that aligns with only one of them) within the discourse context of a story. Inspired by work by Scott et al. (2012), who used a similar method to investigate theory-of-mind rather than word recognition, we presented children with sequential sentences of a coherent story while two naturalistic scenes were displayed visually, one matching the spoken sentence and one not. This method provides a felicitous context in which to investigate children’s response to common properties of spontaneous speech, such as reduction and repeated mentions, which might seem unnatural in a more minimal context.

Using the story-guided looking method, we conducted two pre-registered experiments testing children’s ability to recognize reduced-pronunciation variants of familiar words. In Experiment 1, we tested a sample of 18- to 24-month-olds in a within-participants design, comparing performance on clear pronunciations, reduced pronunciations in repetition contexts (first a clear instance, then a reduced pronunciation in the following sentence), and reduced pronunciations in no-repetition contexts. We expected a sample of adults tested in the same procedure to be adept at recognizing reduced pronunciations regardless of context, and predicted that infants would need more support, relying on helpful repetition across successive sentences (e.g., Schwab & Lew-Williams, 2016) to facilitate recognition of the reduced pronunciations. Indeed, reduced pronunciations frequently follow clearer pronunciations in natural discourses (e.g., Fowler & Housum, 1987; though see Swingley, 2022), suggesting that such an experimental result could reflect adaptation to a frequent pattern in children’s experience. In Experiment 2, which is ongoing, we test a sample of 3-year-olds in the same three conditions—clear pronunciations, reduced pronunciations in repetition contexts, and reduced pronunciations in no-repetition contexts—to further reveal the developmental progression in listeners’ ability to understand pronunciation variability.

## Experiment 1

### Method

#### Participants

Participants were 32 18- to 24-month-olds (mean age = 21;18, 44% female). Participants were recruited primarily through study advertisements emailed to their parents on our behalf by a local children’s hospital. Once their parents expressed interest in the study, infants were eligible to participate if they were typically developing, with no reported ear or hearing problems, had been born full-term, and were being raised in a monolingual English environment (at least 75% English exposure, based on parental estimate). An additional 11 infants were tested but excluded from the final sample because of equipment failures (n=1), concurrent ear infection (n=1), or because they failed to contribute at least 3 trials to each experimental condition (n=9). In keeping with our pre-registration, we excluded any experimental trials where infants failed to fixate the images for at least 2/3 of a second in the analysis window (367 ms to 2000 ms from target-word onset), as well as any trials where the parent or child talked over the speech stimuli.

To establish a baseline and confirm the intelligibility of our stimuli to mature language users, we also collected pilot data from 12 native English-speaking adults, recruited from a university subject pool (undergraduates in psychology or related courses). The procedure for these participants was exactly the same as for the infant participants, except that adult participants were compensated in course credit rather than monetarily, gave informed consent themselves, and were told that this was an infant study, the task being to look at the pictures and follow along with the story.

#### Materials

We tested clear and reduced pronunciations of 12 target words (apple, bottle, doll, fish, ball, shoe, cup, sock, bear, car, book, and keys) that are frequent in speech to children and easily depicted. On each test trial, participants heard a sentence using one of these target words (e.g., “Cheryl takes the *apple* out of the fridge”), while viewing two pictures, one of which matched the spoken sentence. In both the clear and reduced conditions, the target word appeared sentence-medially, so as to keep position in utterance constant. (Utterance-final position, e.g., “Look at the *apple*”, is a less felicitous context for phonetic reduction.) As is standard in language-guided looking studies, items were paired such that the same two objects always appeared together (e.g., apple and bottle).

To create the clear pronunciations, we asked a female native English speaker to hyperarticulate the target word in the sentence, emphasizing the word prosodically and clearly realizing each of its sounds. For the reduced pronunciations, we asked the same speaker to produce the target word and its surrounding context more casually, with less clear realization of the individual sounds and no prosodic emphasis on the target word. Audio editing was used to remove pops/clicks from the recording and adjust the amplitude of waveform. The same speaker also produced the audio material for the filler/story trials, which separated pairs of experimental trials and advanced the overall story (of one character, Cheryl, who wants to go outside, but is delayed by various chores and interactions with another character, Elmo).

The visual materials were naturalistic scenes (e.g., a photo of a young woman’s hand grabbing an apple or a bottle inside a fridge), presented side by side on a gray computer screen (34.7 × 26.0-cm LCD). Each image had an area of 9800 square pixels, and paired images were always of the same orientation (portrait, landscape, or square). All of the visual stimuli were custom-made for the study. Rather than using existing images, we staged, took, and minimally edited photographs as needed to match the text of the story. The story was designed to be engaging to young children and to create a coherent sequence of events from the experimental trials.

To present the stimuli, we used the Experiment Builder presentation software in combination with the EyeLink 1000+ automatic eyetracking system (reported accuracy of .5°), sampling monocularly at 500 Hz. The eyetracker functioned using a camera just below the computer screen, and required no head restraint, using a sticker with a high-contrast pattern on the infant’s forehead to localize the head in space.

#### Procedure

Parents gave informed consent for their child to participate after hearing a verbal explanation of the experimental procedure. Infants were then tested sitting on their parent’s lap, facing a computer display in a dimly lit testing room. Thus, parents remained with their child throughout the experiment. To prevent parents from biasing children’s responses, we asked parents to wear a visor or opaque glasses covering their eyes so that they could not see which image was on which side of the screen.

**Figure 1:**
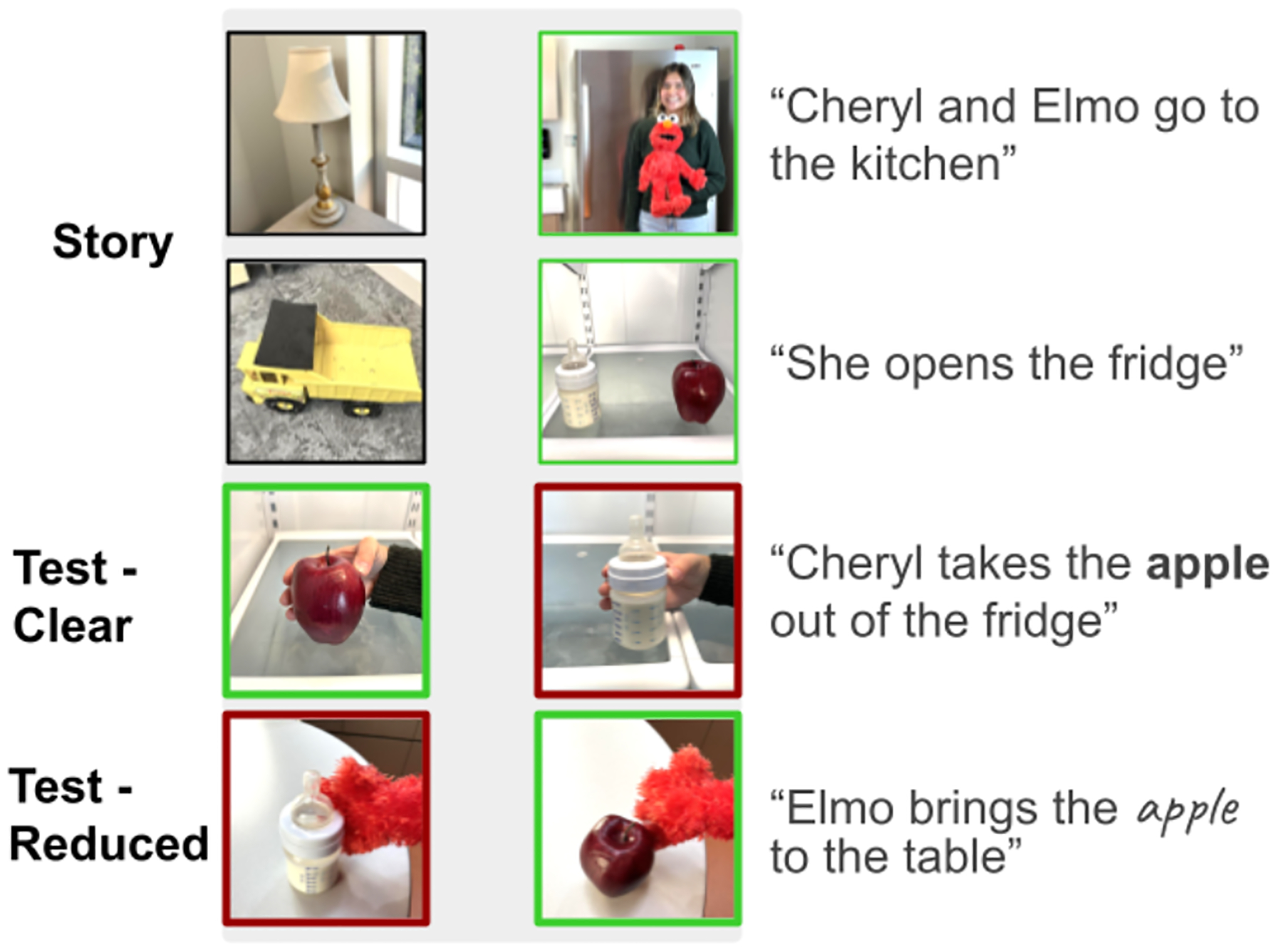
Study Design. On each trial, participants heard a sentence of a story that matched one of two pictures on the screen (light green outline) but not the other. We analyzed looking to the target on pairs of test trials to determine how well infants recognized reduced pronunciation variants of familiar words and whether this depended on the preceding context.

During each trial of the experiment, participants heard a pre-recorded sentence of a story, produced in the manner of spontaneous child-directed speech by a female native English speaker. After two introductory trials with a single image on the right or left side of the computer screen, each trial featured two scenes displayed side by side, only one of which matched the spoken sentence (Figure 1). The correct target image appeared on the left and the right equally often, in pseudorandom order, with the constraint that the target appeared on the same side for at most two trials in a row.

Critically, the story contained 12 pairs of test trials (grouped into repetition and no-repetition pairs) where the two scenes were nearly identical except for a particular named object to probe word recognition. In “repetition” test trial pairs, the first trial in the pair contained a clear instance of a particular word (e.g., “Cheryl takes the **apple** out of the fridge”) and the second trial contained a reduced pronunciation variant of the *same* word (e.g., “Elmo brings the (reduced) apple to the table”). In “no-repetition” test trial pairs, the first trial used the same carrier phrase to reference a *different* object in the story (e.g., “Cheryl takes the **bottle** out of the fridge”) so that the reduced pronunciation variant in the second trial (“apple”) was *not* preceded by a clear instance of the same word.

Within each presentation order, left vs. right target side was equally likely: overall; among the clear test trials; among the reduced test trials; given repetition vs. no-repetition; and given target image side recurrence (same target image side as previous trial vs. not). Additionally, “reduced, repetition” vs. “reduced, no-repetition” test trials were equally likely overall, and by target image side recurrence. Across presentation orders, which words appeared as the targets in which conditions was counterbalanced.

Each test trial pair was separated by 4 filler trials that advanced the story. Including the trials preceding the first test trial pair and following the last test trial pair, there were 78 trials total (24 test trials, 51 filler/story trials, and 3 warm-up or finale trials with only one image).

### Results

Statistical analyses were conducted in R (R Core Team, 2022), using the tidyverse (Wickham et al., 2019), lme4 (Bates et al., 2015), and emmeans (Lenth, 2023) packages. Proportion target looking was computed as the amount of time spent looking to the target image divided by the amount of time looking to either the target or the distractor image during the analysis window, which was defined as any time after the start of speech for the filler/story trials and between 367 and 2000 ms after target word onset for the experimental trials.

#### Adult Pilot

As expected, participants in the adult pilot experiment performed extremely well in this task. In the filler/story trials, the proportion of time spent looking to the correct, target image (out of time spent looking to the target or the unrelated distractor image) was significantly better than chance (*t*(11) = 16.52, *p* < 0.001). Thus, adults were able to follow along with the story, based on the speech or the continuity of the visual elements.

To analyze the data from the experimental conditions, we used the following mixed effects logistic regression model: proportion_target_looking ~ condition (clear; reduced, repetition; or reduced, no-repetition) + location_at_onset + salience + (condition + salience + 1 | subject) + (1 | item). *Condition* was Helmert-coded to measure 1) the effect of clear vs. reduced (combining the two kinds of reduced trials) and 2) the effect of “reduced, repetition” vs. “reduced, no-repetition”. *Location_at_onset* (sum-coded) is a binary variable representing whether the participant happened to already be looking at the correct image at target-word onset, and *salience* (centered) is a measure of how much each participant liked looking at each object (e.g., average proportion apple looking in each of the 4 trials with an apple image, in the period before the speech started.)

**Figure 2:**
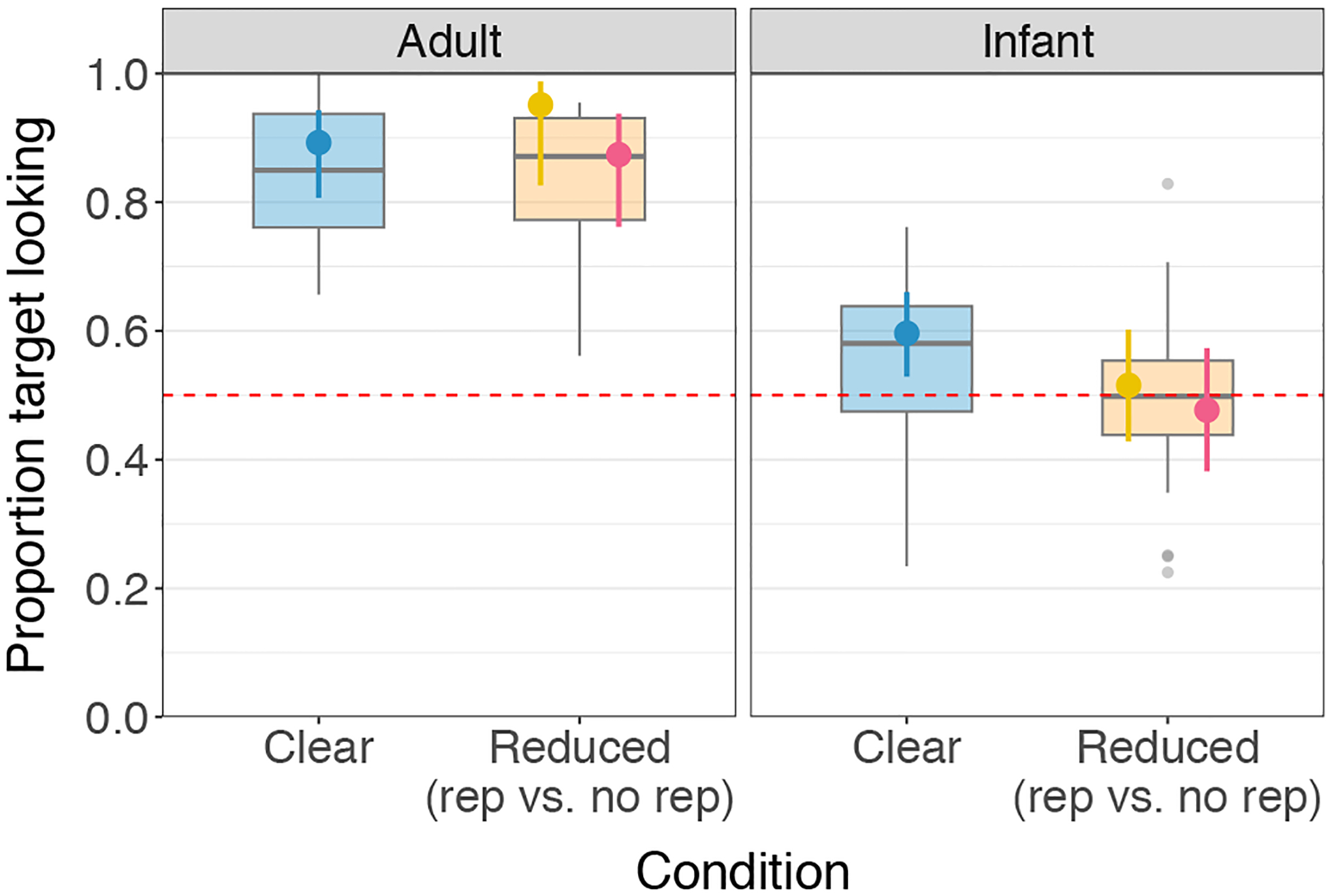
Adults’ and infants’ target looking by condition. For infants, target looking in the clear condition (blue boxplot of subject means) was significantly better than in the reduced conditions (orange boxplot of subject means). The model estimate (solid point with segment indicating model-based 95% confidence interval) for infant performance was significantly above chance in the clear condition (blue) but at chance in the reduced, repetition (yellow) and reduced, no-repetition conditions (pink), with no significant difference between the two.

Using this model, we found that across the three experimental conditions, adults demonstrated target looking significantly above chance (clear: $\hat{\text{P}}=0.89$, 95% CI = [0.81, 0.94], *p* < 0.001; reduced, repetition: $\hat{\text{P}}=0.95$, 95% CI = [0.83, 0.99], *p* < 0.001; reduced, no repetition: $\hat{\text{P}}=0.87$, 95% CI = [0.76, 0.94], *p* < 0.001). (See Figure 2 for a visualization.) The model indicated no significant difference between the clear vs. reduced conditions (β = −0.34, SE = 0.47, *p* = 0.472) or between the reduced, repetition vs. reduced, no-repetition conditions (β = 1.04, SE = 0.78, *p* = 0.180). Thus, regardless of condition, adults were equally and significantly successful at recognizing the target words.

#### Infant Data

The story-guided looking method developed in this study is arguably more complex than other more traditional word recognition tasks. To measure infants’ success at following along with the story, we analyzed their looking behavior in a pre-specified subset of filler/story trials where adults looked at the correct image more than 75% of the time (all but 5 filler/story trials). In these trials, infants also looked at the target image at significantly above chance rates (*t*(31) = 22.04, *p* < 0.001). This result is important for the method, since it demonstrates that switching back and forth between looking the left and the right image as the story progresses is not too difficult or confusing for children in this age range.

We used a mixed effects logistic regression model with same formula described above to analyze infant performance in the experimental trials. We found a significant advantage for the clear over the reduced pronunciations (β = 0.41, SE = 0.18, *p* = 0.025), but contrary to our predictions, no significant difference between the reduced, repetition versus reduced, no-repetition conditions (β = 0.16, SE = 0.26, *p* = 0.553). Infants demonstrated successful recognition (above-chance target looking) of the clear pronunciations ($\hat{\text{P}}=0.60$, 95% CI = [0.53, 0.66], *p* = 0.005), but not of the reduced pronunciations (reduced, no-repetition: $\hat{\text{P}}=0.48$, 95% CI = [0.38, 0.57], *p* = 0.639), even when they had just heard a clear instance of the same word in the previous sentence (reduced, repetition: $\hat{\text{P}}=0.52$, 95% CI = [0.43, 0.60], *p* = 0.729). (See Figure 2 for a visualization.)

**Figure 3:**
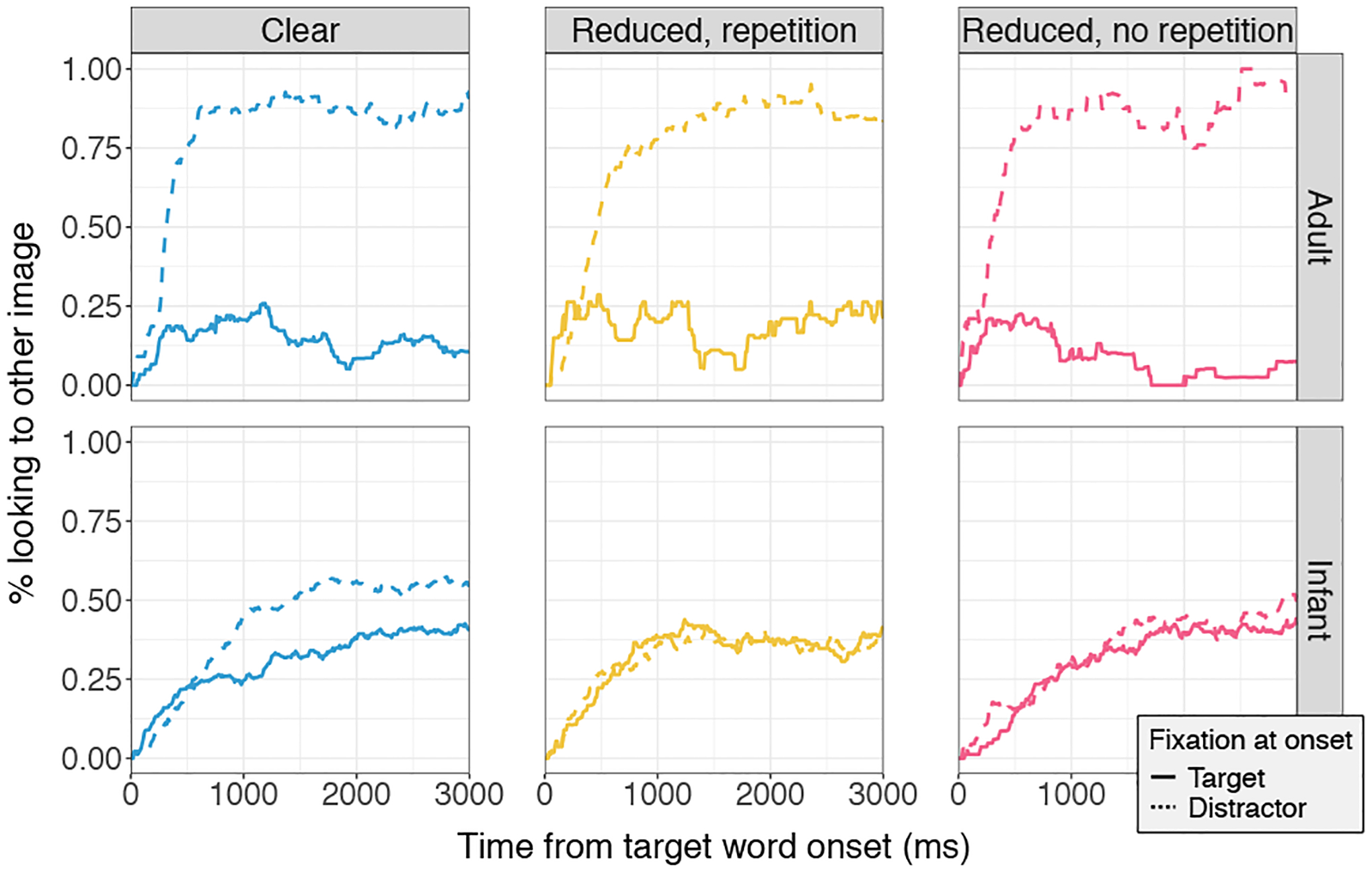
Time course of word recognition for adults vs. infants. Trials are divided according to condition and according to where participants happened to be looking when the target word began (target (solid line) vs. off-target (dashed line)). The x-axis shows time, starting from the acoustic onset of the spoken target word. The y-axis shows the proportion of trials on which participants were, at that moment, looking away from their initial gaze location.

Figure 3 shows the time course of recognition for the infant and adult participants. For the adults (top panel), if the participant happened to already be looking at the target image at target-word onset (solid lines in the figure), then there tended to be little switching to the opposite (distractor) image (i.e., low % looking to other image) at later time points, compared to trials where the participant started off-target (dashed lines in the figure), in which looking to the opposite (target) image later on was more common. In other words, adults heard the target word and looked (or continued to look) at the corresponding target image, regardless of trial type, in line with the statistical analyses. Perfect performance would be shown by the target-onset lines remaining at zero (no defection from the target) and the distractor-onset lines rising to one (always rejecting the distractor). For the infant participants, a divergence can be visually observed only in the clear-pronunciations condition. For both of the reduced conditions, infants switched which image they were looking at over time but in a manner unrelated to the speech they heard, suggesting, in line with our analysis of overall proportion looking, that infants did not recognize the target word in either of the reduced conditions. Whether they heard “(clear) **bottle** … (reduced) apple” or “(clear) **apple** … (reduced) apple”, they did not recognize the reduced pronunciation, despite recognizing the same words in clear speech.

In a second set of analyses (also pre-registered), we investigated potential effects of age or vocabulary size in our sample. Adding age (centered at 21.5 months, measured continuously in days but transformed to months by dividing by 30.42) to the mixed effects logistic regression model described above, we found a significant main effect of age on target looking (β = 0.14, SE = 0.06, *p* = 0.019), with older children showing more target looking overall, but no significant interaction with either of the condition contrasts (age*clear vs. reduced: β = −0.16, SE = 0.12, *p* = 0.174; age*reduced, repetition vs. reduced, no-repetition: β = −0.05, SE = 0.17, *p* = 0.778). Adding vocabulary (mean centered, measured using the MB-CDI Words and Sentences parental checklist for children’s productive vocabulary (Fenson et al., 1994)) to the model instead of age, we found no significant main effect of vocabulary on target looking (β = 0.31, SE = 0.39, *p* = 0.437) and no interaction with either of the condition contrasts (age*clear vs. reduced: β = −0.69, SE = 0.77, *p* = 0.373; age*reduced, repetition vs. reduced, no-repetition: β = −1.21, SE = 1.12, *p* = 0.278). This lack of interaction between condition and either age or vocabulary suggests that within our 18- to 24-month-old sample, children of different ages or different vocabulary sizes experienced the same advantage for clear over reduced pronunciations, and the same non-effect of repetition for the reduced pronunciations.

## Experiment 2

Experiment 1 revealed that while adults are adept at recognizing reduced pronunciations in both repetition and no-repetition contexts, toddlers fail to recognize reduced pronunciations reliably. 18- to 24-month-olds’ target looking for reduced pronunciations was at chance, even in the reduced, repetition condition (first a clear instance, then a reduced pronunciation in the following sentence), which we had expected to support recognition. If 18- to 24-month-olds can only recognize very clear pronunciations of familiar words, when does comprehension of more reduced pronunciations begin, and how do children first resolve this problem which would seem to be essential to everyday communication? To investigate the developmental progression in listeners’ ability to understand pronunciation variability, we conducted a second experiment with a sample of 3-year-old children.

### Method

#### Participants

Participants in the current sample were 17 35- to 39-month-olds (mean age = 37;28, 47% female). Ongoing work is completing the pre-registered sample size of n=44, selected based on a power analysis using the R package mixedpower (Kumle et al., 2021) and trial-level data from Experiment 1. An additional 3 children were tested but excluded from the final sample because they failed to contribute at least 3 trials to each experimental condition. Recruitment procedures and inclusion criteria were the same as those for Experiment 1.

#### Materials

Experiment 2 used the same audio and visual materials prepared for Experiment 1 with a few simplifying modifications. First, the fish image, which was low in salience (rarely fixated before the speech started) compared to its competitor the doll image for infants in Experiment 1, was edited to increase the size of the fish, and the image preview time (before the speech started) for all word recognition test trials was increased from 1000 ms to 2000 ms. Second, to reduce the burden posed by the filler trials, which advance the story without testing recognition of a particular target word, the last filler trial before each pair of test trials was modified to have a single image presented centrally (requiring no decision), and the number of filler trials was decreased from 4 filler trials between each pair of word recognition test trials to only 3 filler trials. To keep the story the same, the spoken sentence for each deleted filler trial was presented immediately before/after the sentence from an adjacent trial, such that some filler trials featured two spoken sentences of the story.

#### Procedure

The procedure was the same as in Experiment 1 except that for 3-year-olds, the parents were not blinded to image side but instead sat in a separate chair next to, but not touching, the child.

### Results

Following our pre-registration, we used a mixed effects logistic regression model including all the same terms as in Experiment 1 except the by-participant random slope for salience, which was not included because in Experiment 1 we were not able to estimate this term independently of the other random effects and salience is only a control predictor rather than the key independent variable of theoretical interest. The full model formula was proportion_target_looking ~ condition (clear; reduced, repetition; or reduced, no-repetition) + location_at_onset + salience + (condition + 1 | subject) + (1 | item).

**Figure 4:**
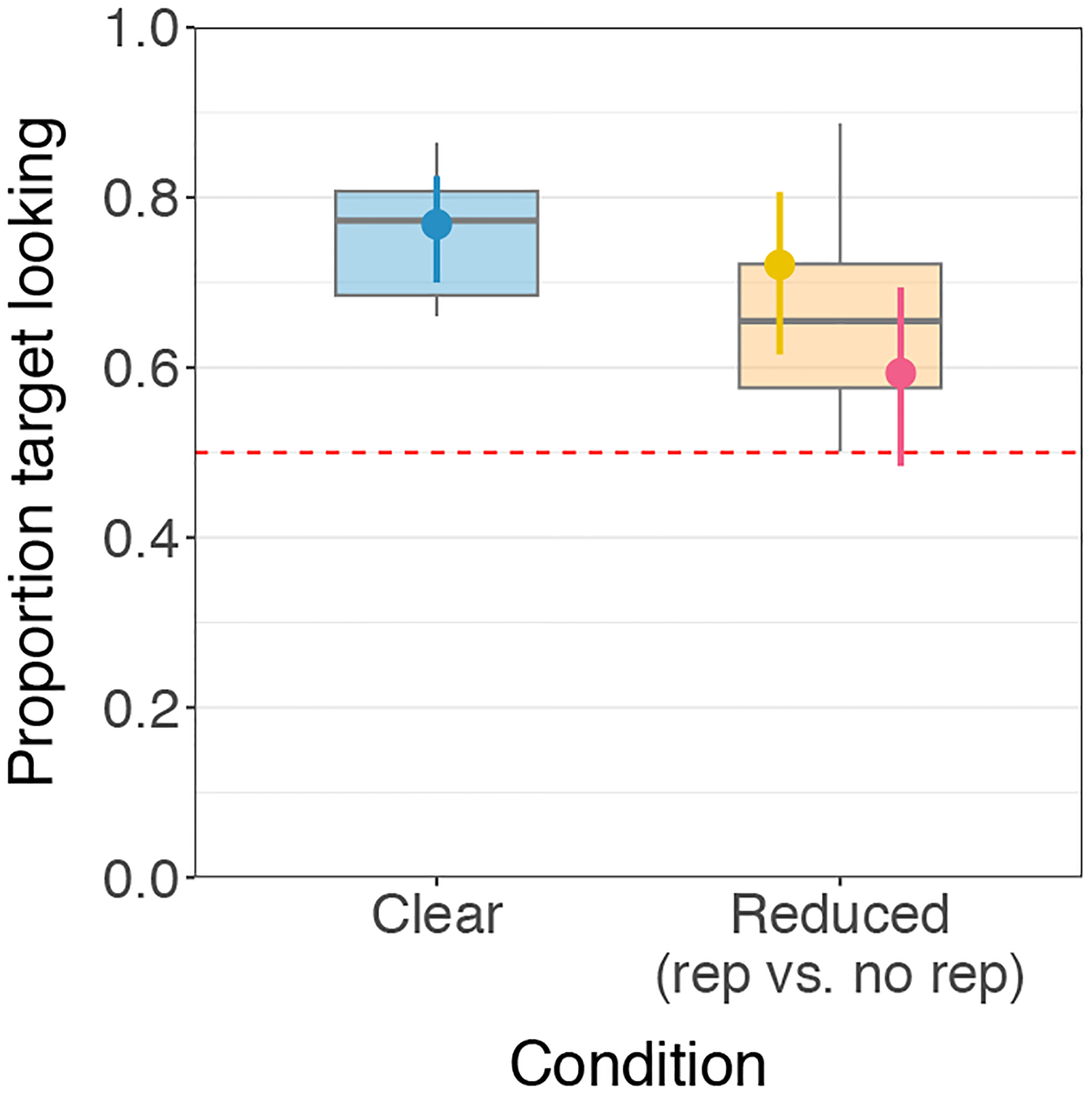
3-year-olds’ target looking by condition (current n=17 out of 44). For 3-year-olds, target looking in the clear condition (blue boxplot of subject means) was significantly better than in the reduced conditions (orange boxplot of subject means). The model estimate (solid point with segment indicating model-based 95% confidence interval) for performance was significantly above chance in the clear (blue) and reduced, repetition condition (yellow), and marginally above chance in the reduced, no-repetition condition (pink), with a marginally significant advantage for reduced, repetition over reduced, no-repetition.

Results for the current sample are shown in Figure 4. Among the 3-year-olds tested so far, we find a significant effect of clear vs. reduced (β = 0.54, SE = 0.24, *p* = 0.027), with more target looking in the clear condition compared to the reduced conditions. We also find more target looking in the reduced, repetition trials compared to the reduced, no-repetition trials, although this difference is only marginally significant in the current sample (β = 0.57, SE = 0.33, *p* = 0.085). Considering each condition estimate, children performed significantly above chance in the clear condition ($\hat{\text{P}}=0.77$, 95% CI = [0.70, 0.83], *p* < 0.001), significantly above chance in the reduced, repetition condition ($\hat{\text{P}}=0.72$, 95% CI = [0.62, 0.81], *p* < 0.001), and marginally above chance in the reduced, no-repetition condition ($\hat{\text{P}}=0.59$, 95% CI = [0.48, 0.69], *p* = 0.094). If these results hold and are found to be significant in the full sample, this would suggest that 3-year-olds are better at recognizing clear pronunciations than reduced pronunciations, but can successfully recognize reduced pronunciations, especially when they occur in repetition contexts.

## General Discussion

In the present study, we investigated young children’s understanding of reduced pronunciation variants of familiar words in repetition and no-repetition contexts. Experiment 1 showed that while our phonetically reduced materials were easy for native English-speaking adults to understand, 18- to 24-month-old toddlers achieved only chance-level performance on the reduced pronunciations, regardless of the preceding context. Experiment 2 data collection is not yet complete (n=17 out of 44 pre-registered), but our interim results suggest that 3-year-olds can recognize reduced pronunciations, particularly in repetition contexts when the preceding sentence contained a clear pronunciation of the same word.

In contrast to previous investigations of infant word recognition, this study employed a new “story-guided looking” method, a more naturalistic variation on “language-guided looking”. In this method, word recognition test trials are embedded in the discourse context of a story rather than in a sequence of independent object labeling events, to provide a felicitous context for properties of normal speech (here phonetic reduction and repetition). Results from Experiment 1 show that children as young as 18–24 months were engaged by and successful at following along with the story in this task, even though the congruent picture switched sides throughout the experiment. Of course, this is a much easier task than succeeding in the test trials, because many of the filler/story trials involved recurring characters or settings (e.g., Elmo or a play room) pitted against a less familiar, and perhaps less visually interesting, distractor scene. Still, children’s success on these trials and success with the experiment length (78 trials in total in Experiment 1) presents a promising picture for future research using the story-guided looking method.

One potential limitation of this method is that while the speech stimuli are naturalistic, they are only produced *in the manner of* spontaneous speech (see Tucker & Mukai, 2022 for discussion). In this study in particular, we relied on our own intuitions, and the speaker’s acting ability, to produce reduced speech stimuli that we thought were representative of the various reduction phenomena that occur naturally (vowel centralization, consonant lenition, coarticulation, decrease in duration, etc.). Future work might attempt to separate these factors out to explore how each contributes to children’s difficulties with reduced speech independently, though existing work suggests that neither duration compression nor vowel hypoarticulation alone completely prevents toddlers from recognizing familiar words (e.g., Song et al., 2010; Zangl et al., 2005). Another alternative would be to investigate children’s understanding of reduced speech from an even more naturalistic perspective: by analyzing where children look in response to parents’ spontaneous speech (e.g., Yu et al., 2021). In our view, these approaches are complementary. One provides more ecological validity, while the other offers substantially more control, allowing researchers to test specific hypotheses while holding other potentially relevant variables constant.

In summary, this work demonstrates a new method for research on children’s understanding of diverse properties of normal speech, and provides new data on 2- and 3-year-olds’ recognition of reduced pronunciation variants, with important implications for theories of language of acquisition. The results from Experiment 2 highlight the role of context, given that 3-year-olds’ success at recognizing a given stimulus seemed to depend on the presence/absence of repetition. In Experiment 1, however, we found no effect of repetition. 18- to 24-month-olds failed to recognize the reduced pronunciation stimuli in either repetition or no-repetition contexts. These results suggest that many instances of words in normal speech are not actually accessible to young children, or at least are not relatable to their clear forms. This challenges the prevailing simplifying assumption that children are able to represent every utterance in terms of its canonical sequence of phonemes, and suggests that, because speech clarity is not necessarily randomly distributed with respect to other aspects of the child’s experience (e.g., Beech & Swingley, 2024), the input to language learning may be inaccurately characterized by corpus-derived counts. Until children can contend with pronunciation variability, the learning problem children face may be very different.
